# COGcollator: a web server for analysis of distant relationships between homologous protein families

**DOI:** 10.1186/s13062-017-0198-x

**Published:** 2017-11-29

**Authors:** Daria V. Dibrova, Kirill A. Konovalov, Vadim V. Perekhvatov, Konstantin V. Skulachev, Armen Y. Mulkidjanian

**Affiliations:** 1 Belozersky Institute of Physico-Chemical Biology, Lomonosov Moscow State University, 119991 Moscow, Russia; 2 School of Chemistry, Lomonosov Moscow State University, Moscow, 119991 Russia; 3 School of Bioengineering and Bioinformatics, Lomonosov Moscow State University, 119991 Moscow, Russia; 4 Department of Physics, Osnabrueck University, 49069 Osnabrueck, Germany

**Keywords:** Clusters of orthologous groups of proteins, Phylogenomic analysis, Comparative genomics, Orthologs, Paralogs, ATP synthase, DNA/RNA helicases

## Abstract

**Background:**

The Clusters of Orthologous Groups (COGs) of proteins systematize evolutionary related proteins into specific groups with similar functions. However, the available databases do not provide means to assess the extent of similarity *between* the COGs.

**Aim:**

We intended to provide a method for identification and visualization of evolutionary relationships between the COGs, as well as a respective web server.

**Results:**

Here we introduce the COGcollator, a web tool for identification of evolutionarily related COGs and their further analysis. We demonstrate the utility of this tool by identifying the COGs that contain distant homologs of (i) the catalytic subunit of bacterial rotary membrane ATP synthases and (ii) the DNA/RNA helicases of the superfamily 1.

**Reviewers:**

This article was reviewed by Drs. Igor N. Berezovsky, Igor Zhulin and Yuri Wolf.

**Electronic supplementary material:**

The online version of this article (10.1186/s13062-017-0198-x) contains supplementary material, which is available to authorized users.

## Implementation

Clusters of Orthologous Groups (COGs) of proteins enable categorizing evolutionary related proteins into more specific groups of orthologs with anticipated similar functionality, which is important for making correct in silico predictions of protein function [1]. Recently a large update of the COG database – including its expansion on the representative set of 711 prokaryotic genomes – was published by Galperin et al. [2]. The COGcollator (Collator of COGs) is a simple online tool that helps to identify evolutionary relations between a particular COG of interest and all the other COGs in the COG database. In addition, the tool enables tracing fusion events that could lead to multidomain (multi-COG) proteins.

### Constructing profiles for each COG

While updating recently the COG database, Galperin et al. [2], did not create COGs for new species, as it had been done previously [1, 3]. Instead, they constructed position-specific scoring matrixes (PSSMs) for existing COGs from 66 earlier published genomes [4] and used them to assign annotated proteins from a larger, up-to date genome database to COGs. Following this approach, we first chose a smaller representative sample with 124 genomes, see (Additional file 1: Table S1) for their complete list. During the sampling procedure we cared for the maximal diversity of taxons, whereby the number of proteobacteria, firmicutes, and actinobacteria sampled was reduced. Second, we created a profile for each COG using the HMMer software [5] based on a multiple sequence alignment of the GOG members that are attributed only to this single COG (this was done to improve the profile selectivity). Proteins were aligned with the Muscle software [6] whereby default parameters were used. This procedure required removal of very small COGs (e.g. those that do not have even three members in the sample of 124 genomes) from our analysis. The resulting set contained 4534 COGs (out of 4631 in the original COG database). It should be noted that the profile quality varies between COGs owing to possible mismatches in multiple alignments of large and poorly conserved COGs. This problem, however, could not be solved without changing the content of original COGs, which was out of the scope of this work.

**Fig. 1:**
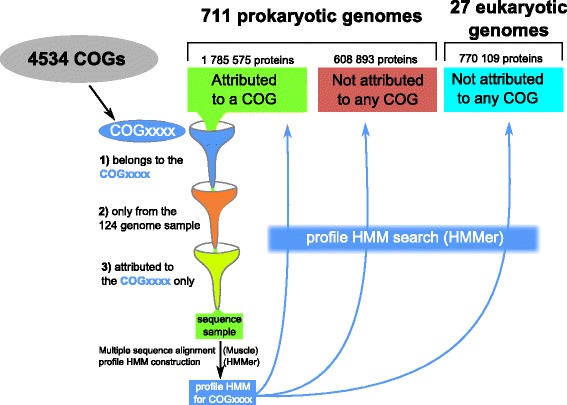
The first part of the COGcollator pipeline. An HMM (hidden Markov model) profile is constructed for each of the 4534 COGs using a subset of sequences that should belong to a particular COG and satisfy two additional conditions, see the main text for details. See Fig. 2 for the detailed description of the step of profile HMM search

Figure 1 shows the pipeline for obtaining hit tables for each COG.

### Visualizing remote evolutionary interaction between COGs

Each profile HMM could be used to search a sequence database yielding a hit table. This table lists the hits, i.e. the regions in any database sequence with a non-random similarity to the profile (above a selected e-value threshold, 10^−5^ in our case). The hits are sorted in a decreasing order by the hit score.

The profiles for each COG were used to search for hits with HMMer software [5] in three protein sequence databases:

1. Proteins from the COG database: totally 1,785,575 proteins from 711 prokaryotic genomes;
2. Proteins from the same 711 organisms, which were not attributed to COGs: totally 608,893 proteins;
3. Eukaryotic proteins from 27 representative genomes (see the Additional file 1: Table S2 for the full list): totally 776,677 proteins.

**Fig. 2:**
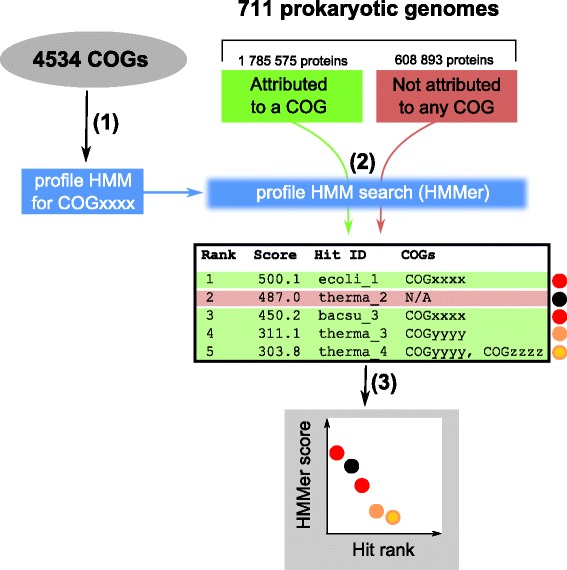
The second part of the COGcollator pipeline (with prokaryotic sequences as an example). **(1)** Profiles are obtained for each COG (see Fig. 1). **(2)** The hit tables for a COG are obtained for each sequence database. **(3)** The prokaryotic hit tables are mixed, ranked and depicted separately from the eukaryotic hit tables (see the main text for the coloring scheme and the Additional file 1: Tables S1 and S2 for the lists of genomes in the 124 prokaryotic and 27 eukaryotic samples, respectively)

As a result, three hit tables are obtained for each COG. The pipeline for visualization of these hit tables is depicted in Fig. 2. For prokaryotic proteins, the hit tables from the first two databases are mixed and ranked together, so that the best hits appear first. This merged hit table is visualized in the COGcollator as a dotted diagram: each dot is a single hit; the dot coloring indicates to which COGs this protein is attributed in the original COG database. The seven COGs, that are most frequently found in the hit table, are rainbow colored with the red color used for the most frequent COG and the violet color for the COG that is ranked seventh, the even rarely occuring COGs are colored grey. The COGs are also distingwished by the dot size, which decreases from the most frequent to the less frequent COGs. The proteins that are not attributed to any COG are shown by the smallest black dots.

If a protein is attributed to several COGs, which is typical for multidomain proteins, a part of the protein sequence is attributed to one COG whereas the other part is attributed to the other COG. Such cases of “COGs fusions” could be informative, provided that they are frequent and thus unlikely to result from a gene misannotation. Accordingly, the respective information is included into the graph that shows overlapping dots for such proteins, whereby the position of the protein on the graph is determined by the more frequent COG denoted by the larger dot (see protein #5 in Fig. 2). The list of COGs on the bottom panel could contain not only the evolutionarily related COGs, but also those COGs that are related through gene fusion (see [7] for a review on the importance of gene fusion cases for function predictions).

### The user interface of the COGcollator and its features

**Fig. 3:**
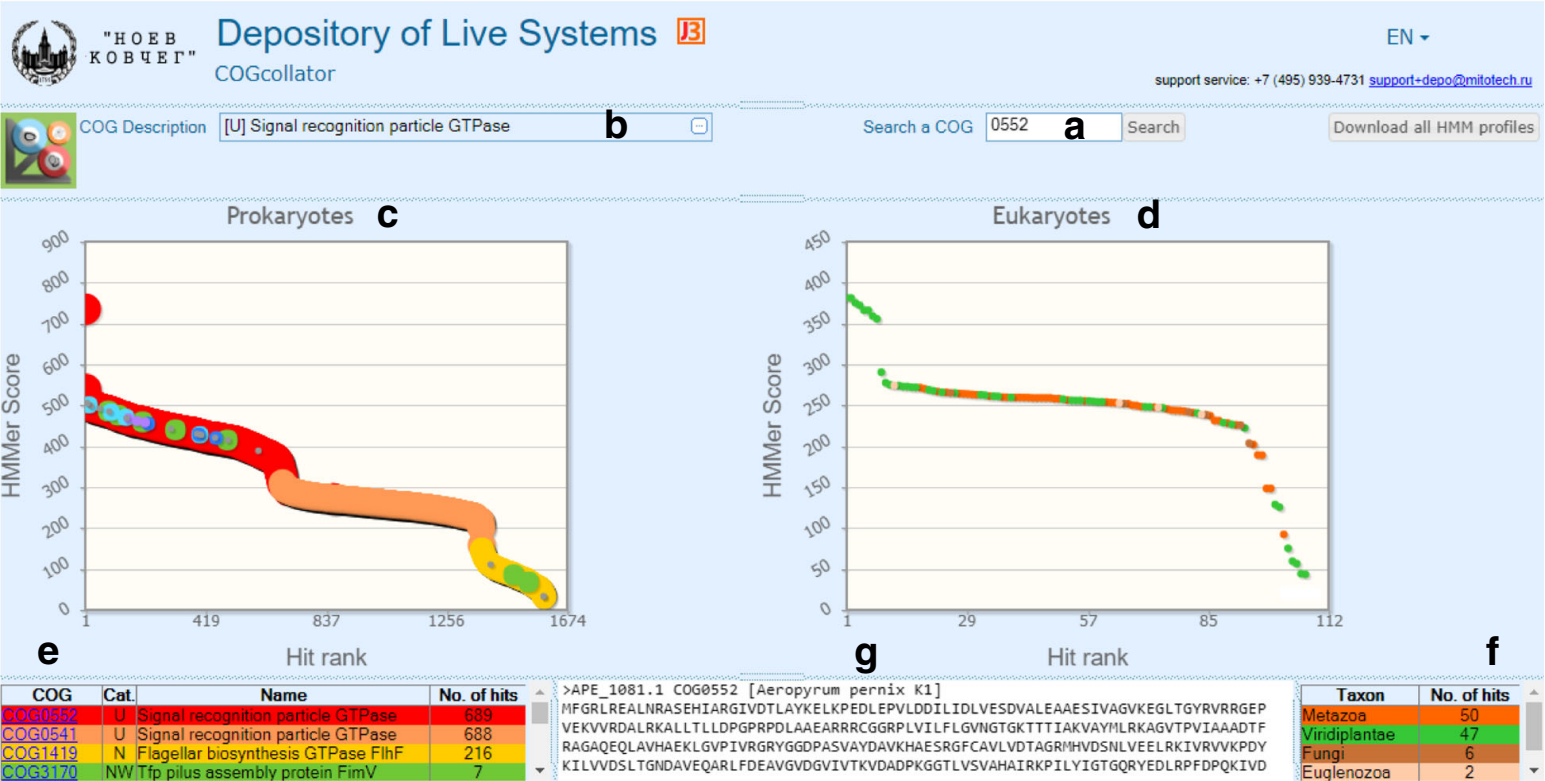
The web interface of COGcollator with the analysis of the COG0552 as an example. See the section “The user interface of the COGcollator and its features” for details

The main panel of the COGcollator is shown in Fig. 3. The user is expected to specify a COG identifier in order to run the server (Panel **a** in Fig. 3). The description of the COG, as taken from the COG database, appears then on the left (Panel **b** in Fig. 3). The analysis results are given on two separate graphs side by side, for prokaryotic and eukaryotic databases, respectively (Panels **c** and **d** in Fig. 3).

The occurrence numbers for each COG are given below the corresponding prokaryotic graph (Panel **e** in Fig. 3), whereby each COG name is hyperlinked to the respective page in the original COG database [2]. Those COGs that are over-represented among the search results are likely to be close relatives of the COG of interest or may be frequently fused with it. While prokaryotic graph is colored as described in the previous section, the eukaryotic graph (Panel **d** in Fig. 3) is colored according to the taxonomy of the organism to which the given dot/protein belongs (see Panel **f **in Fig. 3). This graph reports how well the eukaryotic proteins that are homologous to the COG of interest do fit the COG profile. One could expect that the best eukaryotic hits to a prokaryotic profile would be the proteins obtained through the endosymbiosis; for instance, the top eight dots on the eukaryotic graph (Panel **d** in Fig. 3) are plant proteins that cluster with bacteria on the phylogenetic tree (data not shown). The occurrences of representatives of main eukaryotic taxa on the eukaryotic graph are given below it (Panel **f** in Fig. 3). Both graphs are interactive: they can be zoomed in, and two consecutive clicks on the left mouse button would yield a sample of sequences with the hit ranks located between the clicked values. The sample of sequences in the FASTA format is returned to the bottom window (Panel **g** in Fig. 3), ready for further manipulations.

It is important to note that the shapes of prokaryotic and eukaryotic graphs for the same COG query could differ; furthermore, the shape similarity, if even observed, might be only apparent. For example, the prokaryotic graph for COG0552 (Panel **c** in Fig. 3) is clearly separated into three regions: the red region for COG0552 (the signal recognition particle GTPase), the orange region for the paralogous COG0541 (annotated as a signal recognition particle GTPase as well) and the yellow “tail” region for COG1419 (flagellar biosynthesis GTPase FlhF). The eukaryotic graph (Panel **d** in Fig. 3) also contains a “tail” region, however the respective proteins are not the bacterial flagellar proteins FlhF, but just the truncated proteins that are related to the query COG.

### Availability and requirements

- Project name: COGcollator
- Project home page:https://depo.msu.ru/module/cogcollator
- Operating system(s): Platform independent (web server)
- Programming language: Java, JavaScript, HTML5
- Other requirements: Java SE 1.8 or higher, Tomcat 7.0 or higher
- License: GNU GPL
- Any restrictions to use by non-academics: none declared

## Main text

### Application of the COGcollator: Identification of COGs that are related to COG0055 (F_O_F_1_-type ATP synthase, β-subunit)

The synthesis and hydrolysis of ATP by rotary ATP synthases of bacteria, mitochondria and chloroplasts (the F_O_F_1_-type or F-type ATPases/ATP synthases) are catalyzed by a hetero-hexamer composed of alternating homologous α and β subunits [8–10]. The interfaces between these subunits harbor the nucleotide binding sites. The catalytic reactions take place, sequentially, in the three binding sites that are mostly contributed by the amino acid residues of the β subunits, these sites, as well as the β-subunits proper are called the catalytic ones. The three other binding sites, which are mostly built of residues of the α subunits, are non-catalytic [11]. The overall processing of ATP or ADP molecules by the hexameric protein complex requires cooperation between all six subunits, which is described by the binding change mechanism [12]. The catalytic hexamer is connected via the central and peripheral stalks to the membrane-embedded part of the enzyme complex; as a result, the ATP synthesis or hydrolysis in the catalytic hexamer gets coupled to the translocation of cations (protons or sodium ions) across the membrane [8, 10, 13]. The related rotary ATP synthases of archaea (the A_O_A_1_-type or  A-type ATPases/ATP synthases) and vacuolar ATPases of eukaryotes (V-type ATPases) make a separate family of A/V-type ATPases with homologous A and B subunits arranged in catalytic hetero-hexamers [8, 9, 14, 15]. Here, the A subunits harbor the catalytic binding sites. The α and β-subunits of bacterial-type F_O_F_1_-ATP synthase and the functionally corresponding B and A subunits of the A/V-type ATPases, respectively, belong to four different COGs, namely COG0056 (α-subunits of the F_O_F_1_-type ATP-synthase), COG0055 (β-subunits of the F_O_F_1_-type ATP-synthase), COG1156 (B-subunits of the A/V-type ATPase) and COG1155 (A-subunits of the A/V-type ATPase). Furthermore, subunits of other protein complexes are known to be distantly homologous to the subunits of ATP synthases: these are the flagellar biosynthesis ATPase/type III secretory pathway ATPase (COG1157) and the transcription termination factor Rho, COG1158 (see [14] for discussion of evolutionary relations between these proteins). In contrast to the F-type ATP synthases and A/V-type ATPases, the subunits of the flagellar biosynthesis ATPases, coded by the FliI gene in *Escherichia coli* and its orthologs in other bacteria, form a homo-hexamer consisting of six identical subunits [16]. This hexamer is attached by the product(s) of the FliH gene to the basal body of bacterial flagella as a (FliI-FliH_2_)_6_ complex and is somehow involved in guiding partly unfolded flagellin molecules into the inner void of the bacterial flagellum [16].

**Fig. 4:**
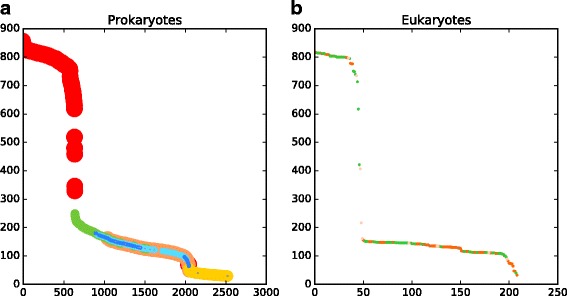
Score vs. hit rank graphs for the COG0055 (the catalytic β-subunit of the F_O_F_1_-type ATP synthase). **a**, prokaryotic sequences; hits are sorted by the score in decreasing order. The dots are colored according to their cognate COGs. The COG0055 (F_O_F_1_-type ATP synthase, the catalytic β-subunit, occurred 647 times) is colored red. The COG0056 (F_O_F_1_-type ATP synthase, the non-catalytic α-subunit, occurred 643 times) is colored orange. The COG1158 (transcription termination factor Rho, occurred 481 times) is colored yellow. The COG1157 (flagellar biosynthesis/type III secretory pathway ATPase, occurred 391 times) is colored green. The COG1155 (A/V-type ATPase the catalytic subunit A, occurred 193 times) is colored cyan. The COG1156 (A/V-type ATPase, the non-catalytic subunit B, occurred 188 times) is colored blue. The COG1372 (intein/homing endonuclease, occurred 8 times) is colored violet. The hits of less frequently occurring COGs are colored grey; **b**, eukaryotic sequences; the dots are colored according to the taxonomy of the corresponding organisms: green for green plants, dark orange for animals, brown for fungi, light orange for other smaller phyla

Figure 4 shows the COGcollator-generated graphs for COG0055 (β-subunits of the F_O_F_1_type ATP synthase). One can see that the aforementioned evolutionarily related groups of proteins appear on the graph. The graph curve has a sigmoid shape, with the inflection point located exactly in the same place where proteins assigned to COG0055 are followed by proteins assigned to the “next” COG, here COG1157 (flagellar biosynthesis ATPase). The correspondence between the inflection point and the “switching” of COGs is not obviously derived from the algorithm of COG construction, which is based on triangles of bidirectional BLAST best-hits in genomes [1, 3]. After the inflection point, the graph curve drops down to a rather smooth plateau with dots assigned to the COG0056 (α-subunits of the F_O_F_1_-type ATP-synthase), COG1156 (B-subunits of the A/V-type ATPase) and COG1155 (A-subunits of the A/V-type ATPase). This region ends with another inflection point, after which only distantly related transcription termination factors Rho (COG1158) are observed.

Surprisingly, the best hits for the catalytic β-subunits of the F_O_F_1_ ATP synthase were neither the catalytic A-subunits, the functional counterparts in the A/V-type ATPases [15], nor the α-subunits of the F_O_F_1_-type ATP synthases, but the flagellar biosynthesis ATPases. Furthermore, when we started the search from COG0056, COG1155 or COG1156, which contain other hexamer-forming subunits of bacterial and archaeal ATP synthases/ATPases, the flagellar ATPases of COG1157 appeared each time as the best hits (data not shown).

**Fig. 5:**
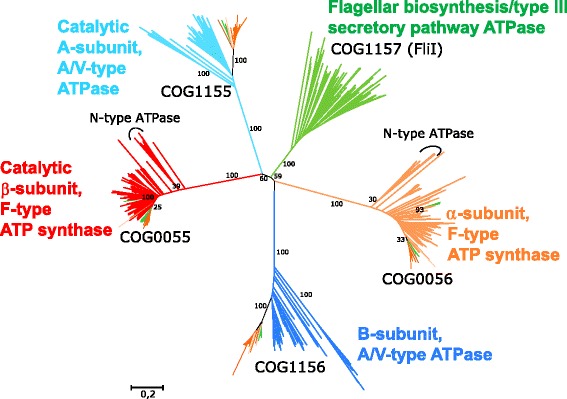
Phylogenetic tree for COG0055 (F_O_F_1_-type ATP synthase, the catalytic β-subunit), COG0056 (F_O_F_1_-type ATP synthase, the non-catalytic α-subunit), COG1155 (catalytic A-subunits, A/V-type ATPase), COG1156 (non-catalytic B-subunits, A/V-type ATPase), and COG1157 (flagellar biosynthesis ATPase). The tree was constructed by using the MEGA 5 software [34] with the neighbor-joining algorithm [35] and JTT matrix for calculation of the evolutionary distances [36], see also (Additional file 2: Figure S1) for the full version of the tree. The sequences were sampled from proteins belonging to the COG0055, COG0056, COG1155, COG1156 and COG1157 from the set of 124 genomes (see*,* Additional file 1: Table S1): totally 368 amino acid sequences were used. We also added sequences belonging to the short representative list of 27 eukaryotic genomes (see*,* Additional file 1: Table S2), totally 196 proteins (with isoforms excluded) were added. Abnormally short (less than 400 amino acids) and long (more than 800 amino acids) sequences were removed, as well as poorly aligned proteins, which resulted in a set of 541 sequences. Conserved blocks were used for the tree construction (totally 391 positions). The branch lengths are in the units of the number of amino acid substitutions per site. Eukaryotic sequences are colored according to the taxonomy of corresponding organisms: orange for animals, brown for fungi, green for plants and light orange for other species. The clades of early-branching N-ATPases [21] are indicated by arcs

We constructed a phylogenetic tree based on the alignment of the α- and β-subunits of the bacterial ATP synthases (COG0056 and COG0055, respectively, as well as corresponding mitochondrial and plastid sequences), A- and B-subunits of the A/V-type ATPases (COG1155 and COG1156, respectively, and eukaryotic sequences of both subunits), and flagellar ATPases of COG1157 (Fig. 5)*.* The tree also shows that the distance between the bacterial flagellar ATPase and any subunit of the A/V-type ATPases and F-type ATP synthases is shorter than the distance between any two subunits of the A/V-type ATPases and F-type ATP synthases. The full version of the tree, which also contains information on neighborhoods of homologous genes, as visualized by the COGNAT software [17], is shown in (Additional file 2: Figure S1).

We also ran a CDD (Conserved Domain Database [18]) sequence search for the α- and β-subunits of the F-type ATP synthase of *E.coli* and for the B- and A- subunits of the A-type ATP synthase of *Methanosarcina mazei*. The results are summarized in Table 1. The hits for the domain cd01136 (Flagellum-specific ATPase/type III secretory pathway) and the respective domain that represents the flagellar ATPase COG1157 in this database always were better (with e-values smaller by about 20 orders of magnitude) than the hits for all other homologous domains (except the domain of the query protein proper). The CDD algorithm, unlike the COGcollator, identifies hits to many profiles for a single sequence.

**Table 1:** Conserved Domain Database [18] sequence search for the α- and β-subunits of the F-type ATP synthase of *E.coli* and the B- and A- subunits of the A-type ATP synthase of *Methanosarcina mazei*

Query	Profile in CDD database				
	Flagellum-specific/type III secretory pathway ATPase	V/A-type ATPase, non-catalytic B-subunit	V/A-type ATPase, catalytic A-subunit	F-type ATP synthase, non-catalytic α-subunit	F-type ATP synthase, catalytic β-subunit
	cd01136	cd01135	cd01134	cd01132	cd01133
ATPA_ECOLI	**1.27∙10** ^**−54**^	2.13∙10^−38^	1.34∙10^−18^	0.0	5.45∙10^−25^
ATPB_ECOLI	**7.82∙10** ^**−68**^	2.13∙10^−32^	6.69∙10^−25^	2.11∙10^−24^	0.0
VATB_METMA	**2.08∙10** ^**−51**^	0.0	3.58∙10^−13^	9.74∙10^−28^	8.29∙10^−23^
VATA_METMA	**2.96∙10** ^**−45**^	3.76∙10^−27^	0.0	1.34∙10^−22^	4.10∙10^−28^

The smallest non-zero e-value for each query is given in bold numbers

By applying the HHpred tool [19] to the same set of sequences we obtained a similar results, with the flagellar ATPases coming out as the best hit for the α- and β-subunits of the F-type ATP synthase of *E.coli* and for the B- and A-subunits of the A-type ATP synthase of *Methanosarcina mazei,* see (Additional file 1: Table S3) for the detailed hit information. Thus our observations got additional support from the data obtained by applying the CDD and HHpred tools.

The appearance of the COG1157 proteins as the best hits for all the four COGs of rotary ATPases was unexpected. One would rather anticipate higher mutual similarity within the “catalytic” β- and A-subunits and/or “non-catalytic” α- and B-subunits, respectively, as the corresponding gene duplication event is widely believed to happen before the separation of bacteria and archaea [20], which is also supported by the trees in Fig. 5 and S1.

The surprising close similarity between the flagellar ATPases and all the four different subunits of rotary ATPases (Figs. 4, 5) might reflect the evolutionary history of these proteins, whereby the so-called N-ATPases, also highlighted in Fig. 5 *,* could serve as an evolutionary link between the flagellar ATPases and the subunits of ATP synthases. Earlier we have identified the N-ATPases as a separate subfamily of bacterial F-type ATPases [21]. While prone to horizontal gene transfer, these complexes are found only in some bacteria and archaea, always in addition to the “main” ATP synthase that is typical for the respective prokaryotic lineage. Since the membrane subunits of these N-ATPases usually (but not always) have complete sets of Na^+^-binding ligands, we have suggested that these enzyme complexes may serve as Na^+^ export pumps in marine and halophilic organisms. And indeed, the N-ATPase of the halotolerant cyanobacterium *Aphanothece halophytica* was shown to translocate Na^+^ ions and to increase tolerance to salt stress in the freshwater cyanobacterium *Synechococcus elongatus* PCC 7942 [22]. Some of the N-ATPases do not have a whole set of Na^+^-binding ligands [21] and appear to translocate protons. Recently, the structure of an unusual heptadecameric membrane ring from such a N-ATPase of *Burkholderia pseudomallei* was resolved by cryo-microscopy [23]. The authors proposed that the N-ATPase of *Burkholderia pseudomallei* serves as a highly efficient proton export pump that helps these pathogenic bacteria to survive in the hostile, acidic environment of phagosomes [23].

The N-ATPases lack the δ subunit of the peripheral stalk; instead, their peripheral *b*-subunit is longer and shows similarity to the E-subunit that makes the peripheral stalk of the archaeal A-type ATP synthase [21]. Recently it has been shown that the FliH subunit, which is involved in the attachment of the (FliH_2_FliI)_6_ complex to the basal body of the flagellum, also structurally resembles the E subunit of the A-type ATP synthase [16], see also (Additional file 3: Figure S2). Figure 5 shows that the subunits of N-ATPases branch closer to the FliI subunits of flagellar ATPases on the phylogenetic tree, as compared to the subunits of the “classical” F-type ATP synthases.

As argued elsewhere [13, 14, 24], the rotary ATP synthases may have evolved from rotary, ATP-driven protein translocases, via an intermediate state of ATP-driven, cellular sodium export pumps. It is tempting to speculate that the distance from the central node in Fig. 5 might correlate with the retention of primitive structural and functional features by the respective protein. Indeed, the flagellar ATPases, the closest ones to the central node(s) in Fig. 5, retain the anticipated primordial function, being involved in the ATP-driven protein translocation [16]. The majority of more remote N-ATPases appear to work as ATP-driven sodium export pumps [21, 22], whereas the most remote subunits of F-type and A/V-tape ATPases are used for synthesis of ATP and therefore seem to be highly derived.

Apparently, homo-hexameric ATPases should have preceded in evolution the hetero-hexameric enzymes. Still, the set of ubiquitous genes, which can be with confidence traced to the Last Universal Cellular Ancestor (LUCA), contains only some genes of hetero-hexameric rotary ATP synthases [14, 25], but not the genes of homo-hexameric flagellar ATPases. To explain this conundrum, we suggest, in accordance with the evolutionary scenarios that we have presented earlier [13, 14, 24], that the emergence of an ancestral, membrane-anchored protein translocase happened before the LUCA. This translocase could possess a catalytic homo-hexameric ring and a peripheral stalk subunit(s) ancestral both to the E-subunit of modern A-type ATPases and the subunits *b* and δ of bacterial F-type ATP synthases [14]. A version of this enzyme complex could attain the ability to use ATP for exporting sodium ions out of the cell [13, 24, 26], which would be important for keeping the K^+^/Na^+^ ratio over unity within the cell [24, 26]. The ion export could be initially driven by hydrolysis of six ATP molecules by homo-hexameric rings of catalytic ATPase subunits (six ATP molecules per turn are believed to be also used by the Rho helicase of COG1158 [27]). The tree in Fig. 5 indicates that the catalytic part of the ion pumping enzyme became hetero-hexameric, as a result of the gene duplication of the catalytic subunit, shortly before the LUCA stage, as suggested earlier [20]. Still, the bootstrap values and lengths of corresponding branches are low, so it could not be fully excluded that the transition to hetero-hexameric enzymes happened just after the separation of domains, independently in bacteria and archaea/eukaryotes. Anyhow, both protein translocases and ATP-driven sodium export pumps may have been present at the stage of LUCA.

The sodium translocating ATPase was inherited by all life lineages. Its genes underwent a duplication in bacteria; this event led ultimately to the separation of more primitive N-ATPases [21] and derived, full-fledged sodium-translocating ATP synthases [30], in which the peripheral stalk subunit split eventually into the subunits b and δ, see the Additional file 2: Figure S2. The homo-hexameric ancestor of flagellar ATPases was, supposedly, lost in the archaeal/eukaryotic lineage, but was retained within bacteria as a part of their flagella. Bacterial flagella, being initially driven by sodium ions [28], appear to emerge as a part of bacterial primordial sodium-dependent bioenergetic machinery [24, 29–31], which also included a hetero-hexameric, sodium-translocating ATPase [13, 24]. With time, the transition to proton-dependent bioenergetics took place in most lineages, following the improvement of membrane tightness and oxygenation of the atmosphere [24, 32].

It is important to mention that the phylogenetic trees previously constructed for the subunits of rotary ATPases (see e.g. [14]) are essentially similar to the tree presented in Fig. 5 and (Additional file 2: Figure S1). Still, the central evolutionary position of FliI (COG1157) was neither noticed, nor described. The “basal” position of the COG1157 became evident only from the COGcollator graphs.

### Application of the COGcollator: Analysis of fusions in the superfamily I of DNA and/or RNA helicase (COG1112)

**Fig. 6:**
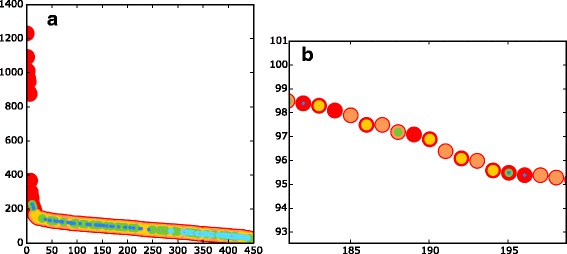
Score vs. hit rank graph for the COG1112 profile in prokaryotic and eukaryotic genomes. **a**, the complete graph; hits are sorted by the score in decreasing order. Dots are colored according to their cognate COGs. The COG1112 (the superfamily I of DNA and/or RNA helicases, occurred 441 times) is colored red. The COG0507 (the ATP-dependent exoDNAse (exonuclease V), α subunit, helicase superfamily I, occurred 164 times) is colored orange. The COG0210 (the superfamily I of DNA or RNA helicases, occurred 54 times) is colored yellow. The COG2852 (very-short-patch-repair endonucleases, occurred 48 times) is colored green. The COG2251 (a predicted nuclease, RecB family, occurred 44 times) is colored cyan. The COG1198 (primosomal protein N′ (replication factor Y) - superfamily II helicase, occurred 38 times) is colored blue. The COG0515 (the serine/threonine protein kinase, occurred 27 times) is colored violet. The hits of COGs with low occurrences are colored grey. **b**, a zoomed region of the graph shown on panel **a**

The superfamily I of DNA/RNA helicases has been earlier identified as a protein family with frequent gene fusions in both bacteria and archaea [33]. Thus, this COG is a good example of a possible bias in the profile HMM, as only a few its proteins are likely to satisfy both conditions required for the COG construction: (1) being present in the 124 genome samples and (2) having a single COG per sequence (see Methods section and Fig. 1 for details). Indeed, only 14 out of 509 (<3%) sequences belonging to the COG1112 were used for the profile creation compared to 99 out of 647 (about 15%) for COG0055 reviewed in the Example #1. However, this profile still identifies 441 (87%) proteins from this COG. A shape of the graph curve is non-sigmoidal, but hyperbolic, with very high scores for the top 7 hits and slow decrease in score for other hits (Fig. 6a). Such a decrease is typical when no distantly related COGs could be found based on the sequence similarity.

Zooming the graph to the scale of individual dots (Fig. 6b) allows inspection of the fusion events. One can see that dots of all other colors are identified on the top of red dots, which indicates that the regions corresponding to the respective COGs are fused to the regions corresponding to COG1112.

It should be noted that the involvement of domains that are readily shuffled between different proteins could lead to blurred results with many grey dots on the graph. These results would still reflect the distribution of such “mobile” domains between the COGs. In such cases, the manual inspection of results —  e.g. by clicking the gray dots on the graph — would be particularly helpful.

## Conclusions

Thus, COGcollator is a reliable tool for tracing evolutionary relationships between the groups of ortologous proteins, as well as fusions of such proteins. With the help of COGcollator and the available COG database [2] the remote relatives can be easily visualized without constructing phylogenetic trees.

## Reviewers’ comments

### Reviewer’s report 1: Dr. Igor N. Berezovsky, Bioinformatics Institute (A*STAR)

***Reviewer 1*** *: This MS is a result of tedious analysis complemented by the visualization options. I do not see neither scientific, not technical advances that would justify publication in Biology Direct.*

**Authors’ response**: In our paper we introduce a web-based tool that allows the user to establish relations between different COGs. A comparable web tool is currently absent in the field. The tool, as correctly noted by the Reviewer, is complemented by visualization options that allow comparison of up to seven COGs simultaneously. Our tool allows fast screening of many targets even by a non-specialist in computational biology. Therefore, by using our tool, more users would be able to apply the COG-based phylogenomic analysis in their research.

We have decided to submit the manuscript to Biology Direct because this journal had recently established a special section “Application Notes” for publication of bioinformatics tools. Since many members of the Editorial Board of Biology Direct either curate the COG database or regularly use it in their work, we anticipated getting fair and professional reviews of our manuscript. Such reviews were indeed provided by Reviewers 2 and 3.

It was not our goal to entertain the reader. Therefore we agree with Reviewer that the description of our web tool might appear tedious for someone who is not a fan of the COG-based analysis. However, we cannot agree with the statement that our manuscript does not offer a technical advance. We have benefited from using this tool ourselves, and we hope that it will help the community in facilitating scientific advances.

### Reviewer’s report 2: Dr. Igor Zhulin, the Oak Ridge National Laboratory

***Reviewer 2*** *: In this paper, Dibrova* et al. *present a web based tool, which enables detection of remote homology between protein families. The tool and underlying process for its development are well described; it can be used by anyone who interested in relationships between protein families. The web server has a user friendly interface: a search is initiated simply by typing the COG identifier and the tool returns related COGs ranked by the HHMer score. Another strength of this tool is its speed – results are generated very quickly, so this allows the user to test and analyze a large number of cases. The authors used the tool to identify remote relatives of ATP synthases and convincingly demonstrated (supporting their conclusions using other methods) similarity between the flagellar ATPases and subunits of F*_*O*_ *F*_*1*_ *-ATP synthases. Authors also demonstrated how their tool can be used for analysis of gene fusion events. I do not have any major concerns about the tool or its description. Minor concerns: 1. The tools has certain limitations, although they are inherent to COG entries themselves. For example, COG X might depict the entire multi-domain protein, whereas COGs Y and Z will depict individual domains that are also present in the multi-domain protein depicted by COG X. In this case, COGalyser* 1 *will show distribution of domain families that are certainly related, but not in any particularly meaningful biological order (HMMer score ranking is the only measure). For example, running COG 0643 (Chemotaxis protein histidine kinase CheA) results in the following hits: 1) COG 2198 (HPT (histidine-containing phosphotransfer domain), which is the N-terminal domain of CheA, but is also present as a stand-alone protein in various signaling systems; 2) COG 0745 (DNA-binding response regulator, OmpR family, contains REC and winged-helix (wHTH) domain) – I can only guess that this COG was picked due to the fact that some, but not all CheA proteins contain the REC domain; otherwise there is no relationship between CheA and OmpR type of proteins; 3) COG 0784 (CheY chemotaxis protein) – the same story here, CheY is a version of REC, so it is homologous to a domain, which sometimes (but certainly not always) is present in CheA (our original search query); 4) COG2205 (K* ^+^ *sensing histidine kinase KdpD) – which is a multi-domain protein, which contains the HATPase_c domain, and so does CheA. So, what we see here is not really a meaningful evolutionary relationship (e.g. homology), but rather relationships between proteins and their parts – individually and jointly. Perhaps it should be pointed out in the paper somewhere that relationships between COGs might be complicated, at least in the case of multi-domain proteins.*

**Authors’ response**: We fully agree with Reviewer and are thankful for the illustrative example that he had provided. We have added the following sentence to the revised manuscript in order to clarify limitation of our server in case of LEGO-type proteins: "It should be noted that the involvement of domains that are readily shuffled between different proteins could lead to blurred results with many grey dots on the graph. These results would still reflect the distribution of such "mobile" domains between the COGs. In such cases, the manual inspection of results - e.g. by clicking the gray dots on the graph - would be particularly helpful.".

***Reviewer 2*** *: It would be useful to indicate what criteria were used to select representative genomes for HMM construction (I presume – phylogenetic relationships, to represent as much diversity as possible without a bias toward “more frequently sequenced” phyla?)*

**Authors’ response**: We are thankful to Reviewer for this comment. We added a descriptive sentence to the “Methods” section of the revised manuscript: “During the sampling procedure we cared for the maximal diversity of taxons, whereby the number of proteobacteria, firmicutes and actinobacteria sampled was reduced”.

***Reviewer 2*** *: I think the name of the tool is awkward. This abbreviation eliminates just 2 letters from a full name - COG analyzer, but this makes it sound… well, awkward. I suggest to go with the full name or to come up with a better abbreviation. I recall the original COG searching tool was called COGnitor. If it is no longer in use, perhaps the authors (Tatusov & Koonin) may allow you to use it!*

**Authors’ response**: Following the Reviewer’s suggestion, we have changed the name of our tool to COGcollator (“Collator of COGs”). The new name puts more emphasis on the potency of the tool to collate the COGs. Accordingly, we have replaced the name in the text of the manuscript and in Fig. 3.

### Reviewer’s report 3: Dr. Yuri Wolf, the National Center for Biotechnology Information, NLM, NIH

***Reviewer 3*** *: Dibrova* et al. *introduce a web tool that visualizes the HMM search scores produced by searching a COG profile against a dataset of prokaryotic and eukaryotic sequences. The web service is up and running, the procedure behind it is adequately described and is scientifically rational, which qualifies this manuscript for publication as an Application Note.*

**Authors’ response**: We are very thankful to Reviewer for testing the web server and analyzing its pipeline.

***Reviewer 3*** *:* *The biggest potential problem with the procedure is the construction of new profiles using an extended range of genomes. Obtaining the new profiles is highly desired because the available diversity of microbial genomes expanded considerably since 2003 when the original profiles were constructed. However the problem is that, in my experience, many COGs are too diverse for an unsupervised alignment using MUSCLE program. Suboptimal alignments of at least some sequences in some COGs are practically inevitable. Since the authors highlight the quantitative aspect of the search results (score* vs *rank plots*), *the utility of the tool hinges on using high-quality profiles. Expert assessment of all alignments is, probably, too expensive, but some semi-automatic validation procedure would probably be useful.*

**Authors’ response**: We fully agree with Reviewer that the quality of profiles varies between COGs. However, a deep filtering of sequences prior to profile construction would actually amend the COGs’ content. In addition, the resulting profile would not fit the removed sequences, and this would raise a question of their proper assignment to this COG. Therefore, at least at the current stage, we prefer to rely on the current COG database release data. Thus, we added the following descriptive sentence to the revised manuscript: “It should be noted that the profile quality varies between COGs owing to possible mismatches in multiple alignments of large and poorly conserved COGs. This problem, however, could not be solved without changing the content of original COGs, which was out of the scope of this work”.

## Additional files

Additional file 1: Tables S1, S2 and S3. Representative list of 124 genomes sampled from the 711 genomes of the current COG database release [2]. **Table S2.** Representative list of 27 eukaryotic genomes sampled manually. **Table S3.** Results of the similarity assessment for the homologs of catalytic β-subunit of the bacterial F_O_F_1_-type ATP synthase by applying the HHpred algorithm [19]. The top hits for the α- and β-subunits of the F-type ATP synthase of *E.coli* and the B- and A- subunits of the A-type ATP synthase of *Methanosarcina mazei* (cf with Table 1) are colored red. (XLSX 29 kb)

Additional file 2: Figure S1. Full phylogenetic tree for COG0055 (F_O_F_1_-type ATP synthase, the catalytic β-subunit), COG0056 (F_O_F_1_-type ATP synthase, the non-catalytic α-subunit), COG1155 (catalytic A-subunits of the A/V-type ATPase), COG1156 (non-catalytic B-subunits, A/V-type ATPase,), and COG1157 (flagellar biosynthesis ATPase) including the diagrams of gene neighborhoods with genes colored according to the provided color code and domain annotation. The gene neighborhoods were visualized with the help of the COGNAT software [17]. See the caption to Fig. 5 for further details. (PDF 466 kb)

Additional file 3: Figure S2. Schematic presentation of sequence similarity between the subunit FliH of bacterial flagella, subunit E of the A/V-type ATPases, subunit *b* of the N-ATPases and subunits *b* und δ of the F-type ATP synthases. (PDF 23 kb)

## Abbreviations

CCD: Conserved Domain Database
COG: Clusters of Orthologous Groups of proteins
HMM: Hidden Markov Model
LUCA: Last Universal Cellular Ancestor
PSSM: Position-Specific Scoring Matrix

## Footnotes

1 The COGcollator web server was called "COGalyser"
