# Supplementary material for: COGcollator: a web server for analysis of distant relationships between homologous protein families
Source: Biol Direct. 2017 Nov 29;12:29. doi: 10.1186/s13062-017-0198-x (PMC5706428; doi:10.1186/s13062-017-0198-x)

#196	COG0636	FoF1-type ATP synthase, membrane subunit c/Archaeal/vacuolar-type H+-ATPase, subunit K
#194	COG0711	FoF1-type ATP synthase, membrane subunit b or b'
#186	COG0055	FoF1-type ATP synthase, beta subunit
#183	COG0056	FoF1-type ATP synthase, alpha subunit
#183	COG0355	FoF1-type ATP synthase, epsilon subunit
#182	COG0224	FoF1-type ATP synthase, gamma subunit
#163	COG0712	FoF1-type ATP synthase, delta subunit
#130	COG0356	FoF1-type ATP synthase, membrane subunit a
#102	COG1155	Archaeal/vacuolar-type H+-ATPase catalytic subunit A/Vma1
#98	COG1156	Archaeal/vacuolar-type H+-ATPase subunit B/Vma2
#90	COG1269	Archaeal/vacuolar-type H+-ATPase subunit I/STV1
#85	COG1390	Archaeal/vacuolar-type H+-ATPase subunit E/Vma4
#85	COG1394	Archaeal/vacuolar-type H+-ATPase subunit D/Vma8
#73	COG1436	Archaeal/vacuolar-type H+-ATPase subunit F/Vma7
#63	COG1157	Flagellar biosynthesis/type III secretory pathway ATPase
#61	COG1527	Archaeal/vacuolar-type H+-ATPase subunit C/Vma6
#48	COG1317	Flagellar biosynthesis/type III secretory pathway protein FliH
#48	COG1766	Flagellar biosynthesis/type III secretory pathway M-ring protein FliF/YscJ
#47	COG1536	Flagellar motor switch protein FliG
#32	COG2882	Flagellar biosynthesis chaperone FliJ
#26	COG3144	Flagellar hook-length control protein FliK
#22	COG3312	FoF1-type ATP synthase assembly protein I
#19	COG3334	Flagellar motility protein MotE, a chaperone for MotC folding

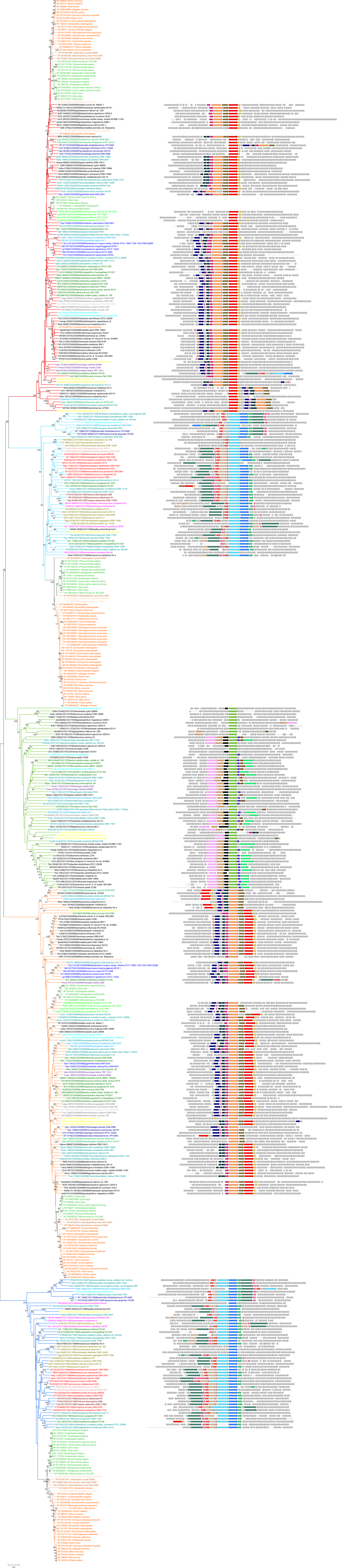

Supplement: Supplementary file 2 — Full phylogenetic tree for COG0055 (FOF1-type ATP synthase, the catalytic β-subunit), COG0056 (FOF1-type ATP synthase, the non-catalytic α-subunit), COG1155 (catalytic A-subunits of the A/V-type ATPase), COG1156 (non-catalytic B-subunits, A/V-type ATPase,), and COG1157 (flagellar biosynthesis ATPase) including the diagrams of gene neighborhoods with genes colored according to the provided color code and domain annotation. The gene neighborhoods were visualized with the help of the COGNAT software [17]. See the caption to Fig. 5 for further details. (PDF 466 kb) [file 13062_2017_198_MOESM2_ESM.pdf]
