# Supplementary material for: COGcollator: a web server for analysis of distant relationships between homologous protein families
Source: Biol Direct. 2017 Nov 29;12:29. doi: 10.1186/s13062-017-0198-x (PMC5706428; doi:10.1186/s13062-017-0198-x)
Supplement: Supplementary file 3 — Schematic presentation of sequence similarity between the subunit FliH of bacterial flagella, subunit E of the A/V-type ATPases, subunit b of the N-ATPases and subunits b und δ of the F-type ATP synthases. (PDF 23 kb) [file 13062_2017_198_MOESM3_ESM.pdf]

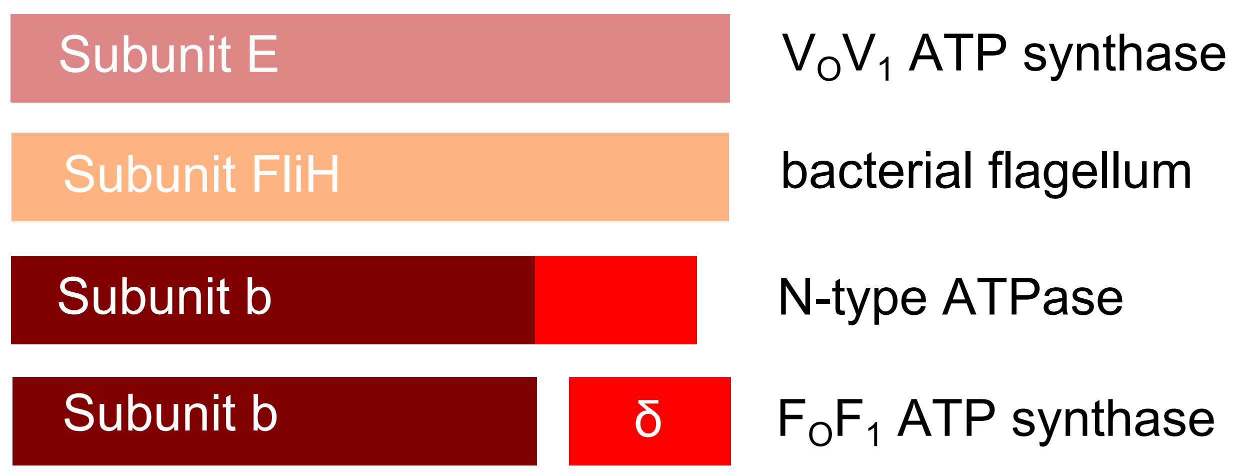
Subunit E
V_OV_1 ATP synthase
Subunit FliH
bacterial flagellum
Subunit b
N-type ATPase
Subunit b
δ
F_OF_1 ATP synthase
